# Nutritional status of a group of polish children with FASD: A retrospective study

**DOI:** 10.3389/fnut.2023.1111545

**Published:** 2023-05-12

**Authors:** Agnieszka Domin, Artur Mazur

**Affiliations:** Institute of Medical Sciences, Medical College, University of Rzeszow, Rzeszów, Poland

**Keywords:** FASD, FAS, ND-PAE, fetal alcohol spectrum disorder, prenatal alcohol exposure, BMI, nutritional status

## Abstract

**Introduction:**

Fetal alcohol spectrum disorders (FASDs) are a leading preventable cause of developmental and psychosocial disorders. Prenatal alcohol exposure can be a cause of growth impairment and metabolic problems. In this study, we analyzed data on the growth, weight, and nutritional status of children with FASD.

**Methods:**

Patients were recruited from the Department of Pediatrics, Pediatric Endocrinology and Diabetology, and the Outpatient Endocrinology Clinic in Rzeszów, Poland. Each person referred for evaluation had a diagnosis of FASD based on the recommendations of Polish experts. The population consisted of 59 subjects with measurements of weight and height, and the IGF-1 level test was performed.

**Results:**

Children with FAS had consistently lower height and weight measurements than children with ND-PAE. In the FAS group, children (<3 percentile) accounted for 42.31%, and in the ND-PAE group – 18.18%. The analysis of the whole group showed the highest prevalence of low body weight (below the third percentile) among subjects with FAS – 53.85%. The prevalence of low body weight and short stature (both parameters <3rd centile) was found to be 27.11% in the whole group. Lower mean BMI values were related to the FAS group (21.71 kg/m^2^) compared to the ND-PAE group (39.62 kg/m^2^). In the study group, BMI below the fifth percentile was found in 28.81% of the children, normal weight (5th-85th percentile) in 67.80%.

**Discussion:**

During the care of children with FASD, a continuous evaluation of nutritional status, height, and weight is necessary. This group of patients is often affected by low birth weight, short stature and weight deficiency, which require differential diagnosis and appropriate dietary and therapeutic management.

## 1. Introduction

Fetal alcohol spectrum disorders (FASD) are one of the leading preventable causes of developmental and psychosocial disorders. Ethanol as a teratogen can produce a highly variable phenotype (1–3). Several diagnostic classifications of FASD are used in parallel around the world. Each of the recommendations used worldwide indicates the need to evaluate four key domains, such as the quantity and quality of alcohol exposure during pregnancy, dysmorphic facial characteristics, prenatal and postnatal growth impairment, and neurodevelopmental disorders (4–7). In Poland, 2 FASD diagnoses were distinguished in the recommendation stated in 2020: fetal alcohol syndrome (FAS) and neurodevelopmental disorder associated with prenatal alcohol exposure (ND-PAE).

Fetal alcohol syndrome is diagnosed based on the presence of several characteristics: (1) deficiency of body weight/height or low birth weight, (2) presence of 3 facial dysmorphia (narrow upper lip, flat fissure, short eyelid crevices), social and neurodevelopmental disorders, confirmed/unconfirmed exposure to alcohol. ND-PAE (neurodevelopmental disorders associated with prenatal alcohol exposure) can be diagnosed in children when exposure is proven and they suffer from neurodevelopmental and psychosocial difficulties. Height or weight deficiency is not necessary for this diagnosis. Children at risk of FASD are a nondiagnostic category that includes children too young for diagnosis or in the process of diagnosis and burdened by prenatal alcohol exposure to further follow-up for FAS, NDPAE, or exclusion of FASD (8, 9).

Both syndromes, FAS and ND-PAE, present characteristics of abnormal cognitive and social development, such as abnormal executive functions, memory problems, reduced motor skills, behavioral disorders, emotions and sensory problems. These impede proper social adaptation, affect the child’s behavior and often also nutritional status. Differences are due to abnormal growth parameters in children with FAS, while in ND-PAE they should be observed less frequently.

In the current study, an analysis was performed on the body weight, height and nutritional status of infants, children and adolescents under the care of the Children’s Endocrinology Clinic and the Outpatient Clinic. These parameters are part of the diagnosis and their disorders require further treatment and management of patients. Finding height deficiency requires a detailed differential diagnosis, including endocrine disorders (10, 11). A detailed perinatal history is necessary, as low birth weight is a risk factor for overweight and obesity, and low height in adulthood, precocious puberty, increased risk of hypertension or carbohydrate and lipid disorders (12, 13). Studies in children with FASD have found that excessive weight gain is correlated with age, with a concomitant reduction in body height centiles (14).

The purpose of this study was to assess the nutritional status of a group of children with FASD. Health professionals can play a key role in the prevention of FASD during pregnancy (15, 16). After birth, the main goal is to identify children with FASD early to prevent secondary problems: extreme low growth or socialization problems (17–19).

## 2. Materials and methods

### 2.1. Study design

This study was conducted from March 2019 to December 2021. The Ethics Committee of the University of Rzeszów approved the study (date of approval: 14 February 2019). All procedures performed in studies involving human participants were in accordance with the ethical standards of the institutional and/or national research committee and with the Declaration of Helsinki of 1964 and its subsequent amendments or comparable ethical standards. Anthropometric data was recorded by the study physicians.

### 2.2. Laboratory analysis

Venous blood samples were tested for IGF-1 and other parameters and collected in the morning between 8:00 and 10:00 a.m., fasting. The blood was then incubated at room temperature for at least 30 min and centrifuged (1,500 × g, 10 min, 4°C). IGF-1 assays were performed by enzyme-amplified chemiluminescent immunoassay (EACLIA) using a laboratory kit (Siemens, #L2KIGF2) on the IMMULITE 2000 System Analyzer. Reference ranges of IGF-1 level are listed in Table 1.

**Table 1:** Laboratory reference ranges of IGF-1 level according to gender and age.

Age	IGF-1 reference level	
	Female	Male
0–3 years	18,2–172 ng/mL	<15,0–129 ng/mL
4–6 years	35,4–232 ng/mL	22–208 ng/mL
7–9 years	56,9–277 ng/mL	40,1–255 ng/mL
10–11 years	118–448 ng/mL	68,7–316 ng/mL
12–13 years	170–527 ng/mL	143–506 ng/mL
14–15 years	191–496 ng/mL	177–507 ng/mL
16–18 years	190–429 ng/mL	173–414 ng/mL

IGF-1 index: calculated as the quotient of the IGF-1 level and the mean value of the IGF-1 standard. IGF-1 has age-specific standards. This parameter was calculated to standardize the result for the entire population and to allow comparison between groups and calculation of correlations between parameters. Other clinical parameters were obtained from the patient’s clinical records.

### 2.3. Participants

#### 2.3.1. FASD group

A single-center cross-sectional study was conducted in 59 children aged 4 months to 16 years and 6 months who were diagnosed with FASD. The patients were recruited from the Department of Pediatrics, Pediatric Endocrinology and Diabetology, and the Outpatient Endocrinology Clinic. Children under two years of age were excluded from the BMI analyses due to the lack of percentile grids for this age group. Informed consent was obtained from all participants or, if they were under 16 years of age, from a parent and/or legal representative. Participation was voluntary.

**Figure 1:**
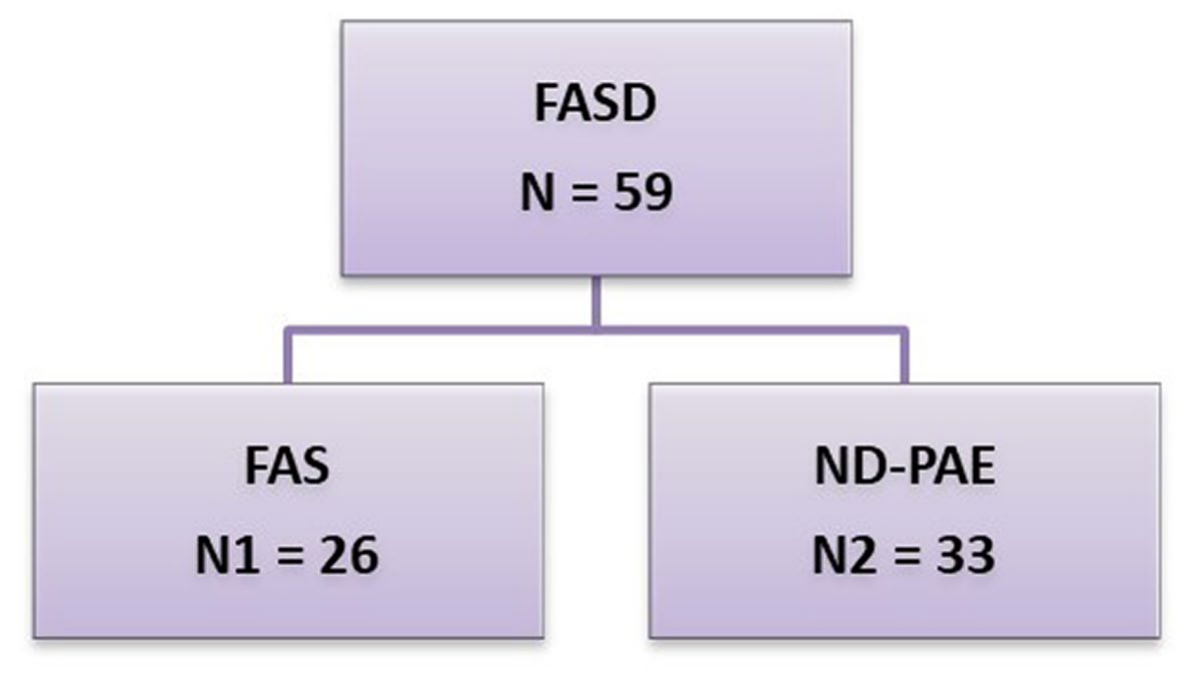
FASD groups by diagnosis. This group was divided into 2 subgroups: N1 – FAS (fetal alcohol syndrome), N2 – ND-PAE (neurobehavioral disorder-prenatal alcohol exposure).

FASD was diagnosed according to the latest guidelines contained in Polish recommendations (8, 9). With respect to guidelines applicable internationally the Polish category ND-PAE includes: pFAS (partial fetal alcohol syndrome) and ARND (alcohol-related neurodevelopmental disorder) (Figure 1).

#### 2.3.2. Healthy controls group

Twenty-three unexposed youth (mean age 7.45 ± 5.12 y, F/M = 7/16) aged 5 months to 17 years were recruited verbally. These participants had confirmed the absence of PAE based on a retrospective interview with the caregiver/parent at the time of blood sampling. These participants had no prior or suspected diagnosis of a neurodevelopmental or other current health problems.

### 2.4. Definitions of BMI and nutritional status

The calculator available on the CDC website was used to calculate the percentile of BMI and the *z*-score for height, weight, and BMI. This tool allows you to calculate the BMI over 24 months of age (20).

For BMI below the 1st percentile, the value of 0.1 was adopted; for values above the 99 percentile, the value was 99.9 (Table 2).

**Table 2:** Weight status category (20, 21).

Weight status category	Percentile range
Underweight	Less than the 5th percentile
Healthy weight	5th percentile to less than the 85th percentile
Overweight	85th to less than the 95th percentile
Obesity	Equal to or greater than the 95th percentile

### 2.5. Statistical analysis

Statistical analysis was performed using Statistica 13.1 PL software (StatSoft Inc., Poland). The distribution of the variables was checked with the Shapiro–Wilk test. Variables whose distribution significantly deviated from normality were analyzed using non-parametric methods. Data were presented as mean ± standard deviation (SD) for variables with a normal distribution and median and interquartile range (IQR) for variables with a significantly different distribution. To assess the differences between groups, Student’s t test and Mann–Whitney U test were used, respectively. Kruskal-Wallis ANOVA was used for multiple comparisons. A *p* value below 0.05 was considered statistically significant. The correlations between the two quantitative variables were tested using the Spearman rank correlation test. Statistical significance was defined as *p* < 0.05.

## 3. Results

A total of 59 FASD patients were included in this study, including 28 boys and 31 girls. FASD patients were classified into 2 groups: FAS and ND-PAE. At the same time, 23 healthy children, including 16 boys and 7 girls, were enrolled in the study. The basic demographic and anthropometric parameters of FASD patients and healthy controls are presented in Tables 3, 4.

**Table 3:** Baseline anthropometric and demographic characteristics of the FASD study group and the control group.

		FASD	Healthy controls	*p* value	*p* variance
Sex (F/M)		31/28	7/16		
Age (years)	Mean ± SD	8.14 ± 3,96	7.45 ± 5.12	0.517	0.123
	Range	0.42–16.5	0.42–17		
BMI percentile	Mean ± SD	31.9 ± 27.48	51.39 ± 27.48	**0.021**	0.542
	Range	0.1–99.9	12.0–99.9		
Weight *z*-score	Mean ± SD	−1.21 ± 1.77	−0.77 ± 0.9	0.318	0.006
	Range	−5.06–2.1	(−2.81)–0.95		
Height *z*-score	Mean ± SD	−0.61 ± 1.44	−0.67 ± 1.07	0.856	0.189
	Range	−3.52−4.14	(−2.35)–1.43		
BMI *z*-score	Mean ± SD	−1.07 ± 1.62	−0.47 ± 1.01	0.161	0.039
	Range	−4.62–2.63	(−2.35)–1.07		
Clinical laboratory markers					
IGF-1 [ng/mL]	Mean ± SD	165.8 ± 100.99	201.97 ± 129.29	0.308	0.244
	Median	132	190		
	Range	28.3–481	53.4–397		
IGF-1 index	Mean ± SD	0.86 ± 0.42	0.86 ± 0.37	0.996	0.701
	Median	0.8	0.74		
	Range	0.28–2.31	0.38–1.47		

Statistically significant differences are bolded. BMI, body mass index; IGF-1, insulin like growth factor-1; comparison between means was analyzed using the Mann–Whitney *U* test or Student’s *t* test.

**Table 4:** Baseline anthropometric and demographic characteristics of the study subgroups FAS and ND-PAE.

		FAS	ND-PAE	Healthy controls	*p* value	*p* value*
Sex (F/M)		14/12	15/16	7/16		
Age (years)	Mean ± SD	7.97 ± 4.67	8.26 ± 3.36	7.45 ± 5.12	0.691	0.697
	Range	1.83–16.5	2.08–13.5	0.42–17		
BMI percentile	Mean ± SD	21.71 ± 27.01	39.62 ± 32.89	51.39 ± 27.48	**0.004**	**0.030**
	Median	12	38	50		
	Range	0.1–78	0.1–99	1–99		
Weight *z* score	Mean ± SD	−2.09 ± 1.74	−0.53 ± 1.48	−0.77 ± 0.93	**0.001**	**0.001**
	Range	(−5.06)–0.99	−2.4–4.14	(−2.81)–0.95		
Height *z* score	Mean ± SD	−1.10 ± 1.42	−0.21 ± 1.35	−0.67 ± 1.07	0.079	**0.03**
	Range	(−3.52)–1.3	(−2.4)–4.14	(−2.35)–1.43		
BMI *z* score	Mean ± SD	−1.77 ± 1.62	−0.52 ± 1.41	−0.47 ± 1.01	**0.007**	**0.005**
	Range	(−4.62)–0.78	(−3.14)–2.63	(−2.35)–1.07		
	Clinical laboratory markers					
IGF-1 [ng/mL]	Mean ± SD	157.71 ± 94.15	172.22 ± 107.3	201.97 ± 129.29	0.614	0.507
	Median	129	137	190		
	Range	28.3–372	71.2–481	53.4–397		
IGF-1 index	Mean ± SD	0.82 ± 0.4	0.89 ± 0.44	0.86 ± 0.37	0.843	0.555
	Median	0.82	0.78	0.74		
	Range	0.29–2.22	0.32–2.3	0.37–1.47		

Statistically significant differences are bolded. BMI, body mass index; comparison between means was analyzed using the Kruskal-Wallis ANOVA test (between FAS, ND-PAE and healthy controls) or the ^*^Mann–Whitney U test (between FAS and ND-PAE).

The average age in the study group (FASD): 8.14 years (in individual groups: FAS – 7.97, ND-PAE – 8.26). In the control group, the average age was 7.45 years. There were no statistically significant differences in the age of the children between the FASD group and the control group (*p* = 0.51). In the FAS and ND-PAE groups, the age of the children at the time of the study did not differ significantly (*p* = 0.78). Subsequently, analyses were performed on anthropometric parameters relating to body height and nutritional status, as well as IGF-1.

### 3.1. Height results

In the FAS group, children with height below the third percentile accounted for 42.31%, while the majority of children with body height below the third percentile were girls 90.91% compared to boys 9.09%. In group ND-PAE, children with height below 3rd percentile accounted for 18.18%, with the largest group being children between the 25th and 50th percentile (27.27%), with a frequency of 50% in both girls and boys (Table 5). No children with body heights above the 90th percentile were observed in the study group. The majority (81.09%) were children with heights below the 50th percentile. In the FAS group: 100% of the patients were below the 75th percentile and 76.93% were below the 25th percentile. The proportion of patients with low height in each group was: (N1: N2) = 11: 6, among girls 10: 3, among boys 1: 3.

**Table 5:** Weight and height percentiles in subsequent groups FASD, FAS, and ND-PAE and healthy controls.

Percentile		FAS Count	NDPAE Count	FASD Count	Healthy controls Count
**<3**	**Height %**	**11**	**6**	**17**	**3**
		**42.31**	**18.18**	**28.81**	**13.04**
	**Weight %**	**14**	**7**	**21**	**1**
		**53.85**	**21.21**	**35.59**	**4.35**
3-10	Height %	4	3		
		15.38	0.09		
	Weight %	5	5		
		19.23	15.15		
10-25	Height %	5	7		
		19.23	21.21		
	Weight %	1	3		
		3.85	9.09		
25–50	Height %	4	9		
		15.38	27.27		
	Weight %	2	5		
		7.69	15.15		
50–75	Height %	2	6		
		7.69	18.18		
	Weight %	3	11		
		11.54	33.33		
75–90	Height %	0	2		
		0	6.06		
	Weight %	0	1		
		0	3.03		
90–97	Height %	0	1		
		0	3.03		
	Weight	0	0		
		0	0		
**3-97**	**Height %**	**15**	**27**	**42**	**20**
		**57.69**	**81.82**	**71.19**	**86.96**
	**Weight %**	**12**	**26**	**38**	**21**
		**46.15**	**78.79**	**64.41**	**91.3**
>97	Height %	0	0	0	0
		0	0	0	0
	Weight %	0	0	0	1
		0	0	0	4.35

The values in the table for the ranges below the 3rd percentile and the 3rd-9th percentile are in bold.

**Figure 2:**
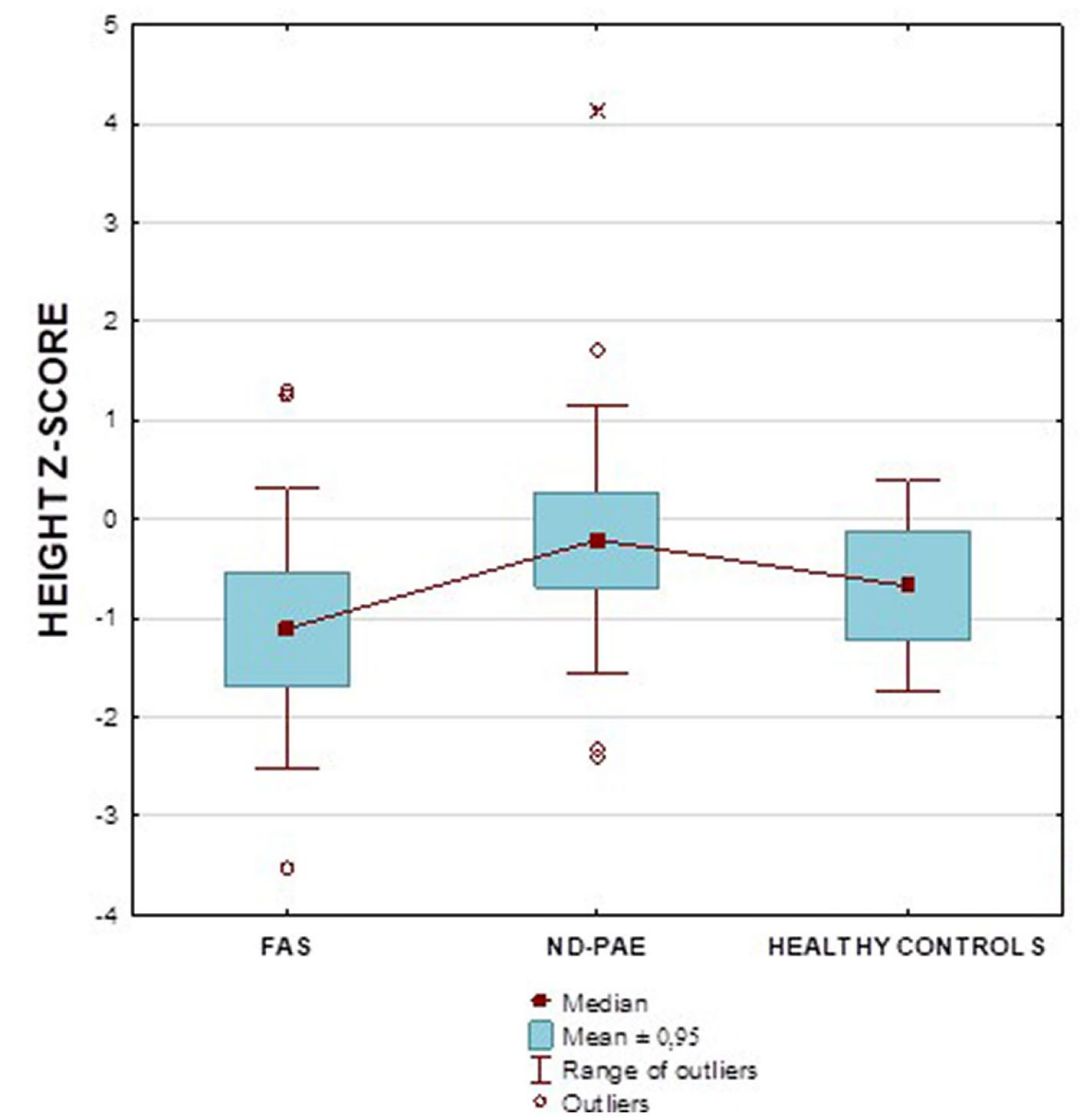
Height *z*-score in patients with FAS and ND-PAE compared to healthy participants.

**Figure 3:**
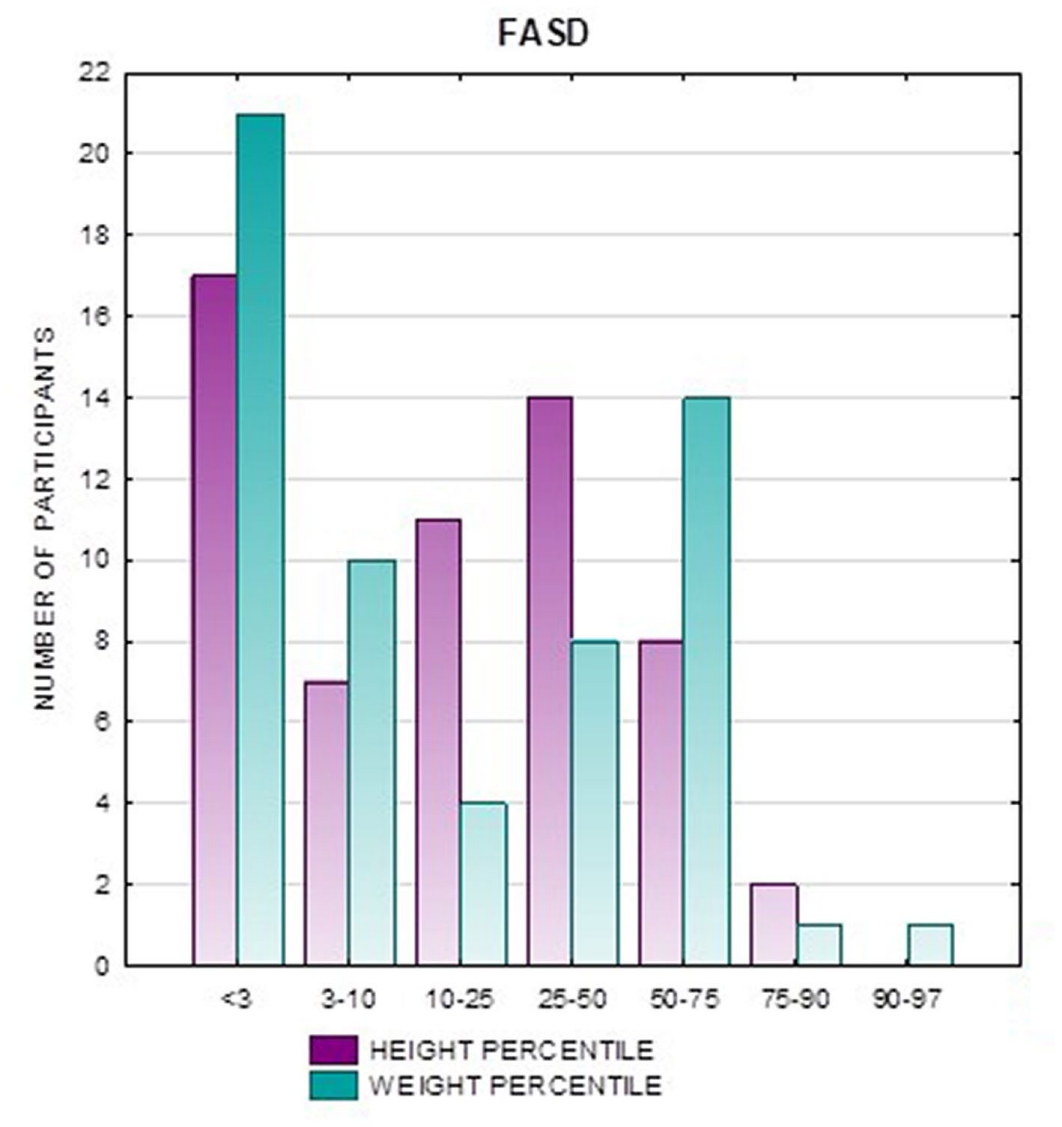
Height and weight percentiles in patients with FASD.

**Figure 4:**
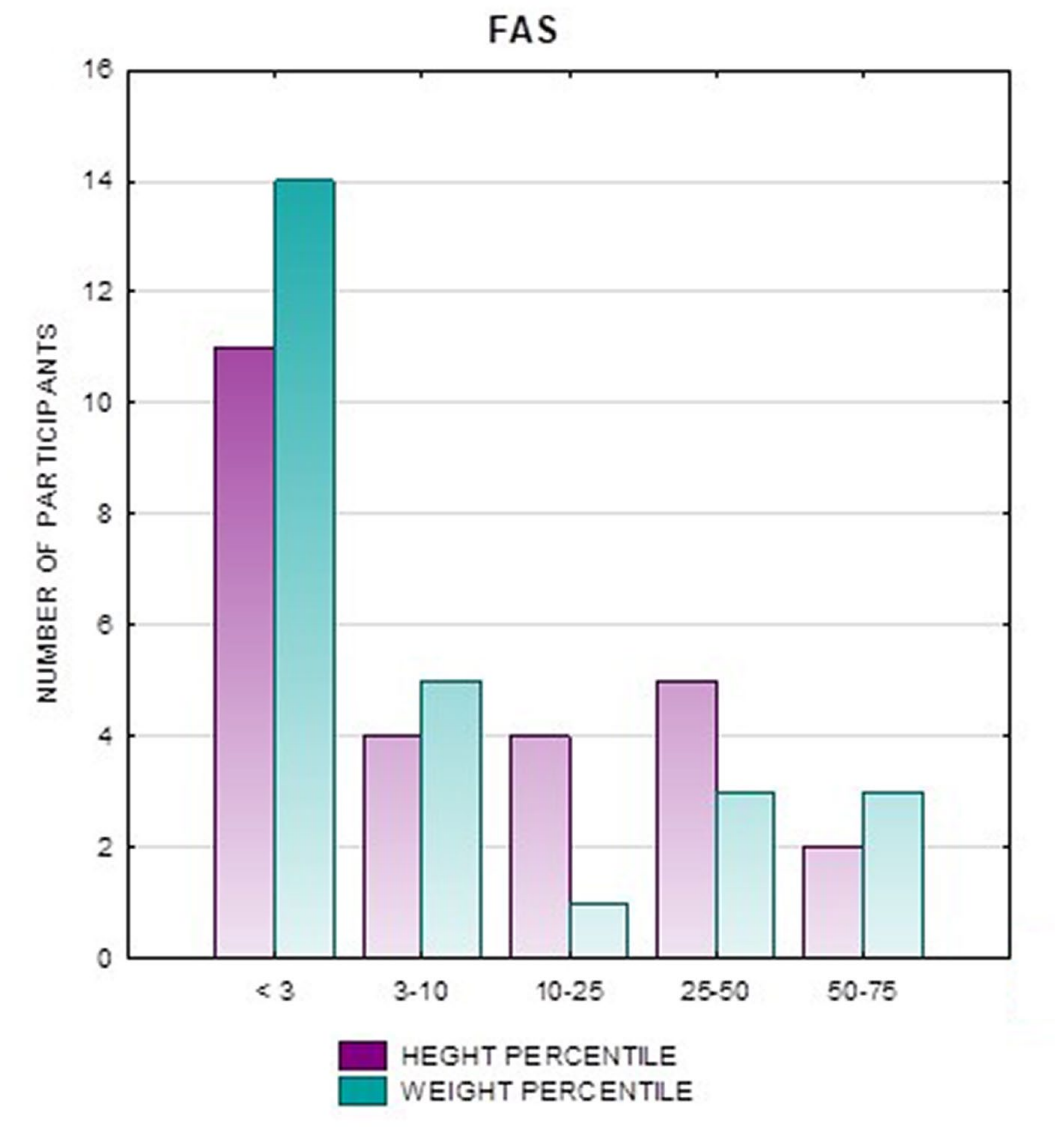
Height and weight percentiles in patients with FAS.

**Figure 5:**
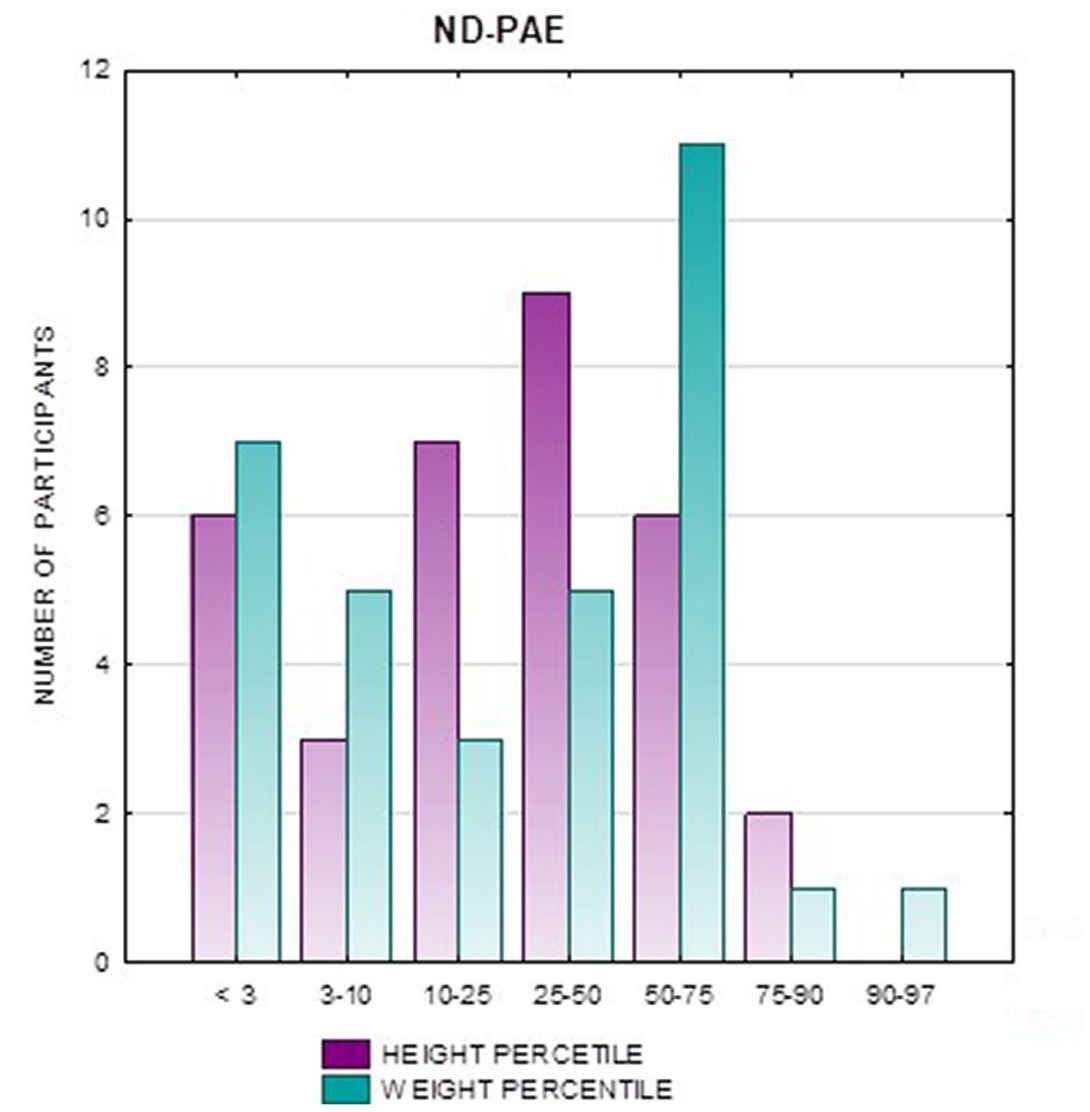
Height and weight percentiles in patients with ND-PAE.

**Figure 6:**
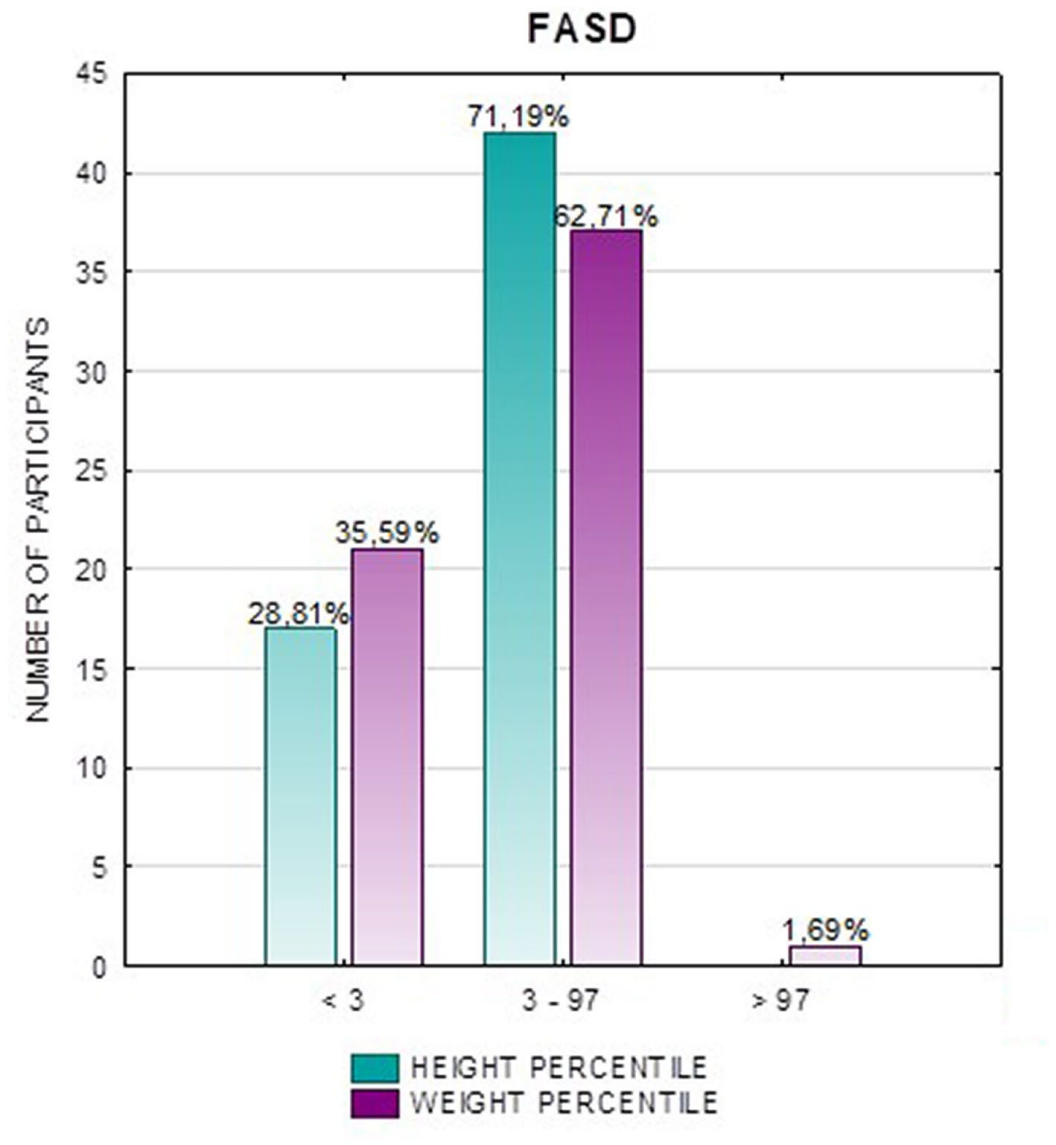
Height and weight deficiency and normal anthropometric parameters in patients with FASD.

**Figure 7:**
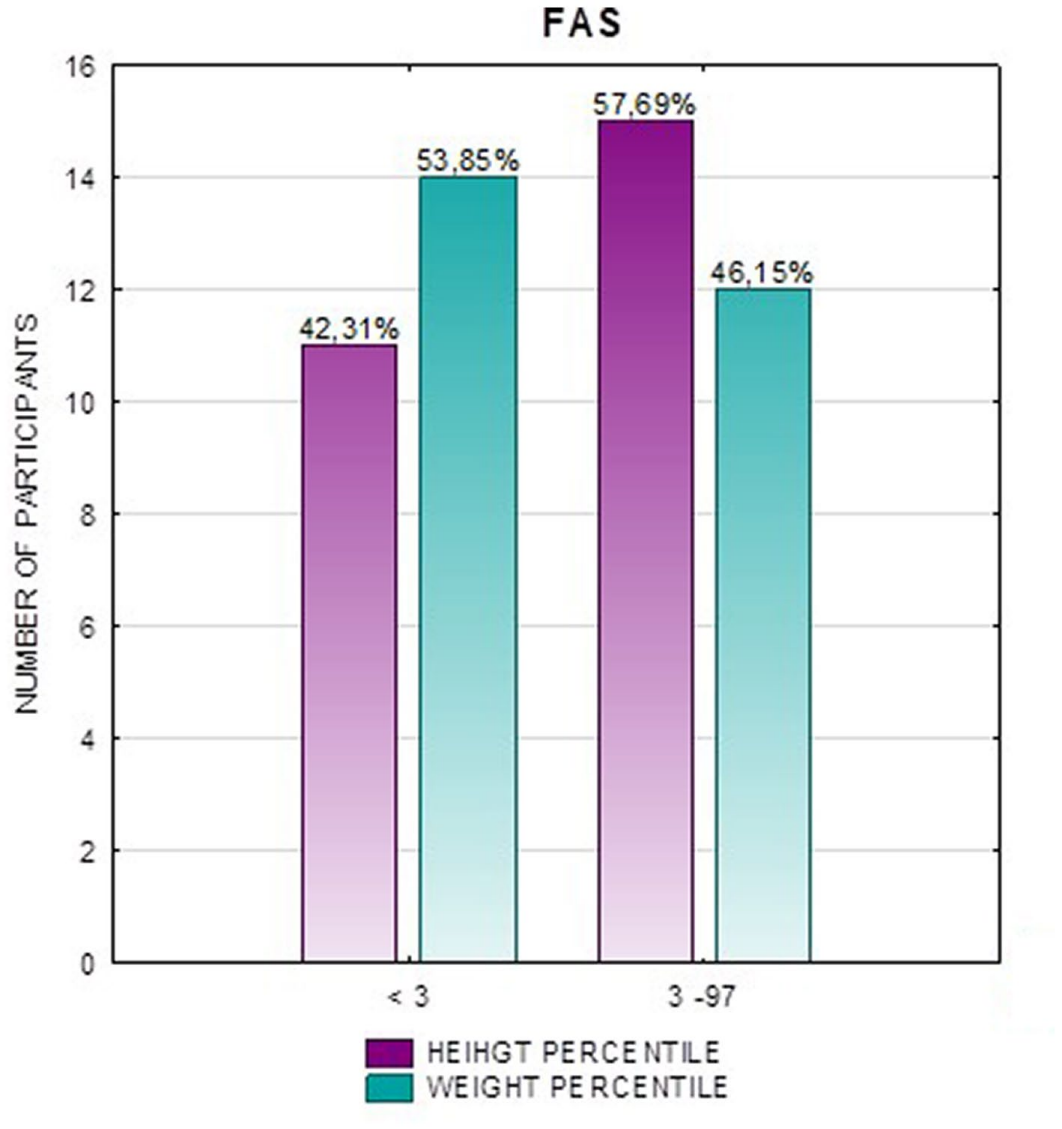
Height and weight deficiency in patients with FAS.

**Figure 8:**
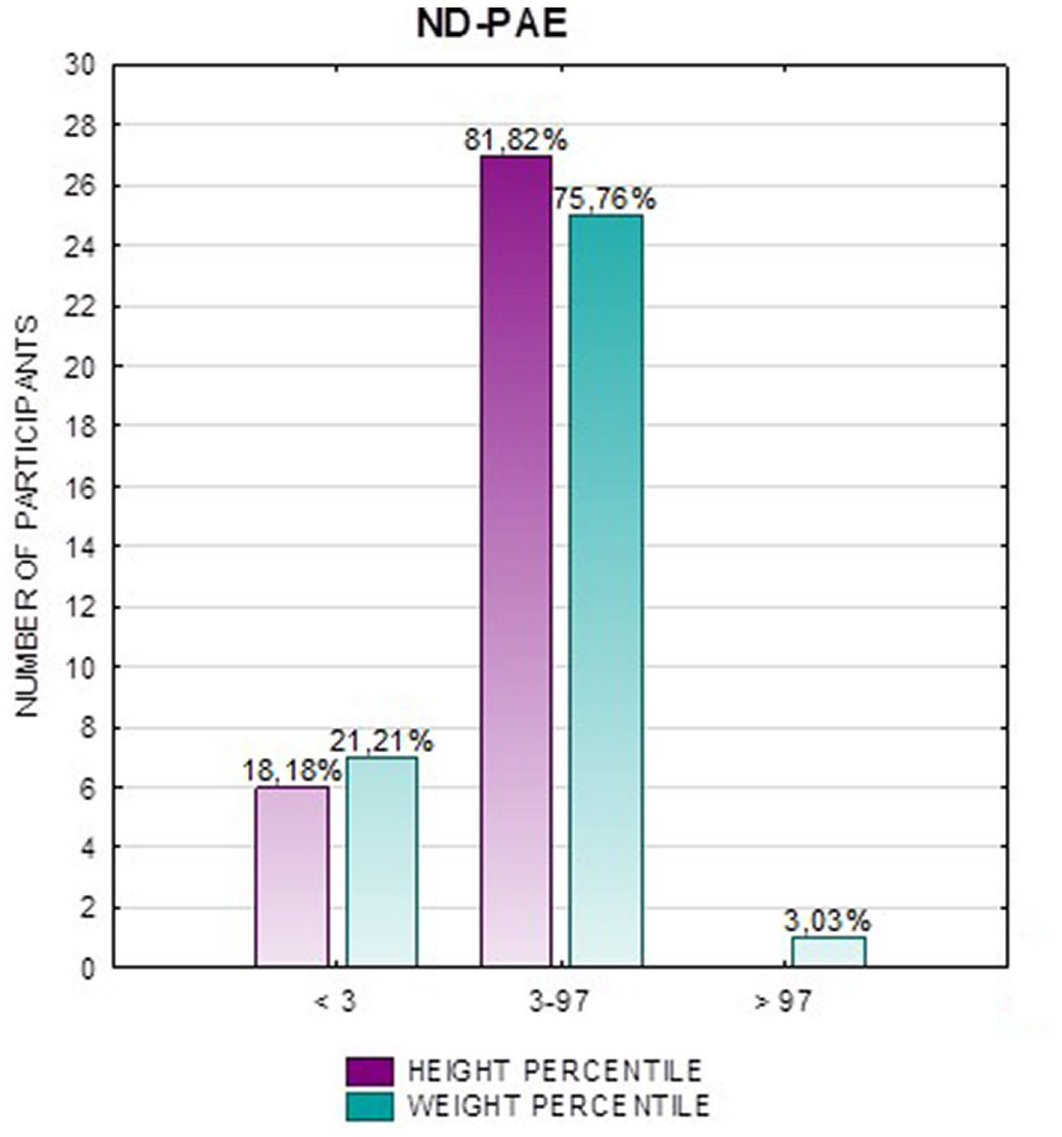
Height and weight deficiency in patients with ND-PAE.

Figure 2 shows the height *z*-score relationships between the FASD subgroups and healthy controls. The other data are presented in Figures 3–8.

### 3.2. Weight results

Analysis of the whole group showed the highest prevalence of low body weight (below the 3rd percentile) among the FAS subjects – 53.85%. In the individual groups, low body weight was observed in the FAS group – 53.85%, and in the ND-PAE group – 22.21%. No children with weight above the 97th percentile were observed in the study group, the majority (95.31%) were children with weight below the 75th percentile (Table 5). In the FAS group, 100% of the patients were below the 90th percentile and 73.08% were below the 10th percentile. Among girls, weight below the third percentile was present in 78.57%, and among boys in 25.0%. In the ND-PAE group, children with low body weight < 3 percentile accounted for 21.21%, the most numerous was the group of children between the 50th and 75th percentile – 33.33%, in this group 60.61% were children below the 50th percentile of body weight. The ratio of patients with low body weight in each group was: N1: N2 = 14: 7, among girls 11: 3, among boys 3: 4. The data are presented in Figures 3–8.

Furthermore, analyses of height, weight, BMI were performed using a calculator from the CDC website in the form of *z*-score results for the FAS and ND-PAE groups and comparisons were made between these groups relative to each other and the control group. The differences between the FAS AND ND-PAE subgroups in terms of weight, height and BMI were statistically significant (*p* < 0.05, Tables 3, 4).

**Figure 9:**
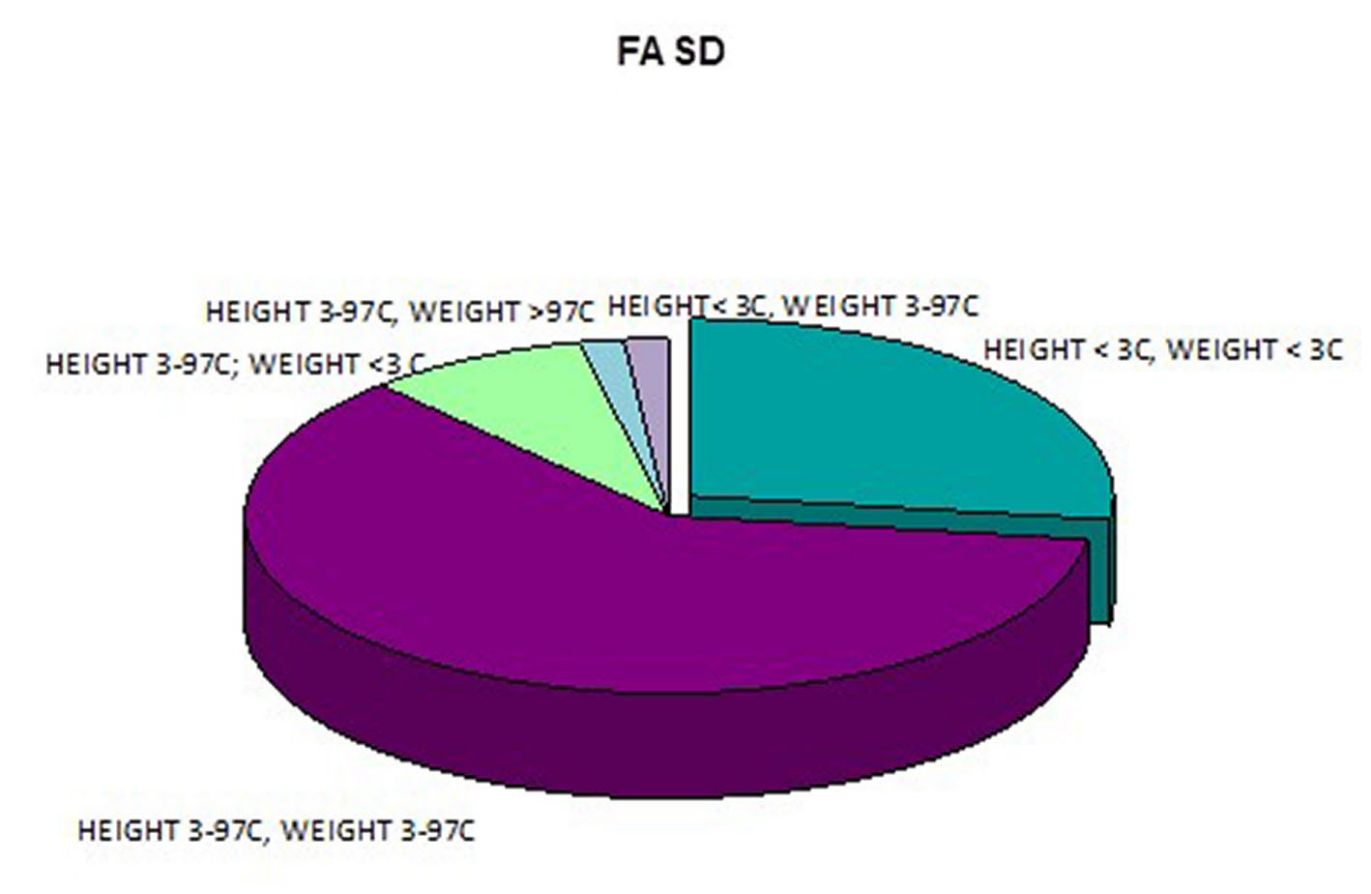
Co-occurrence of weight and height deficiency in patients with FASD.

**Figure 10:**
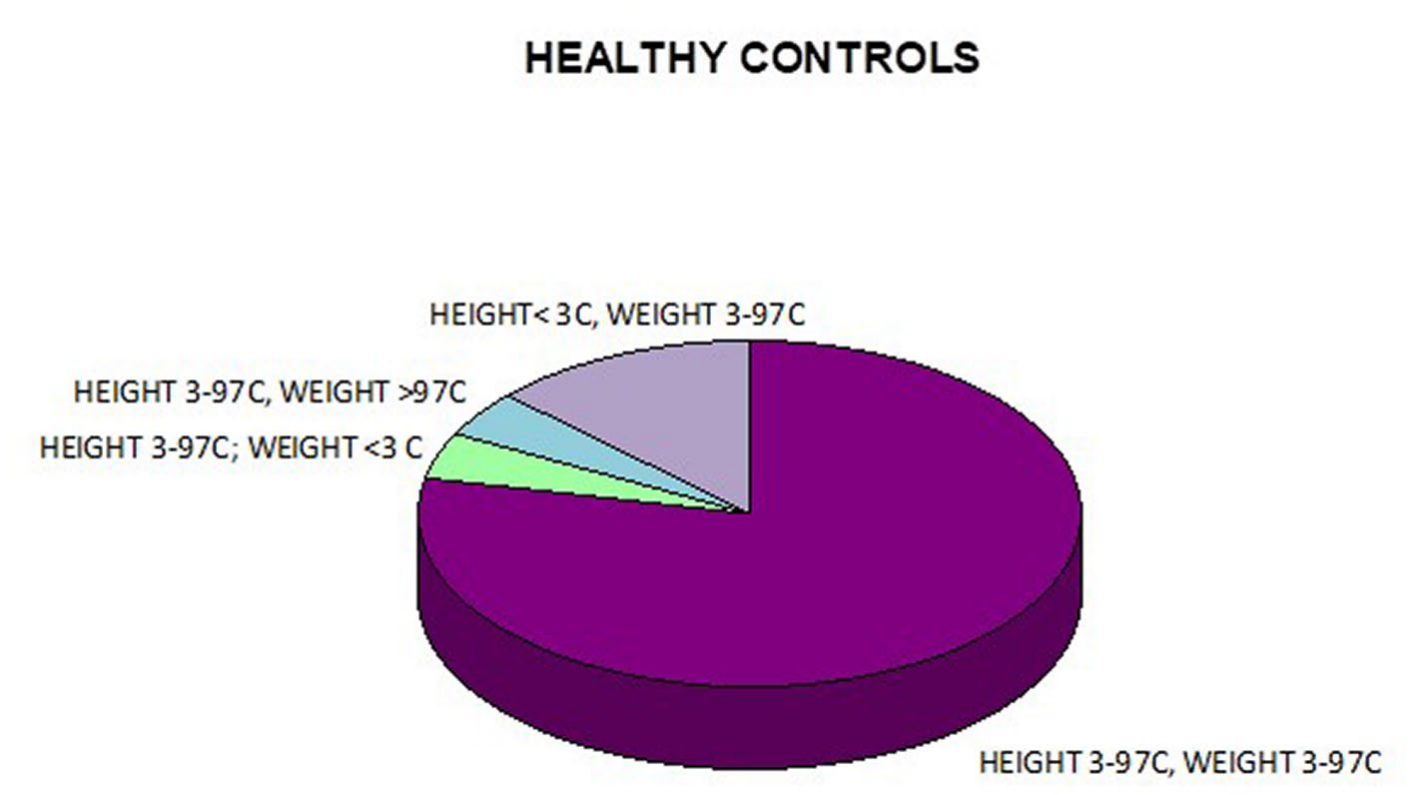
Co-occurrence of weight and height deficiency in healthy controls.

**Figure 11:**
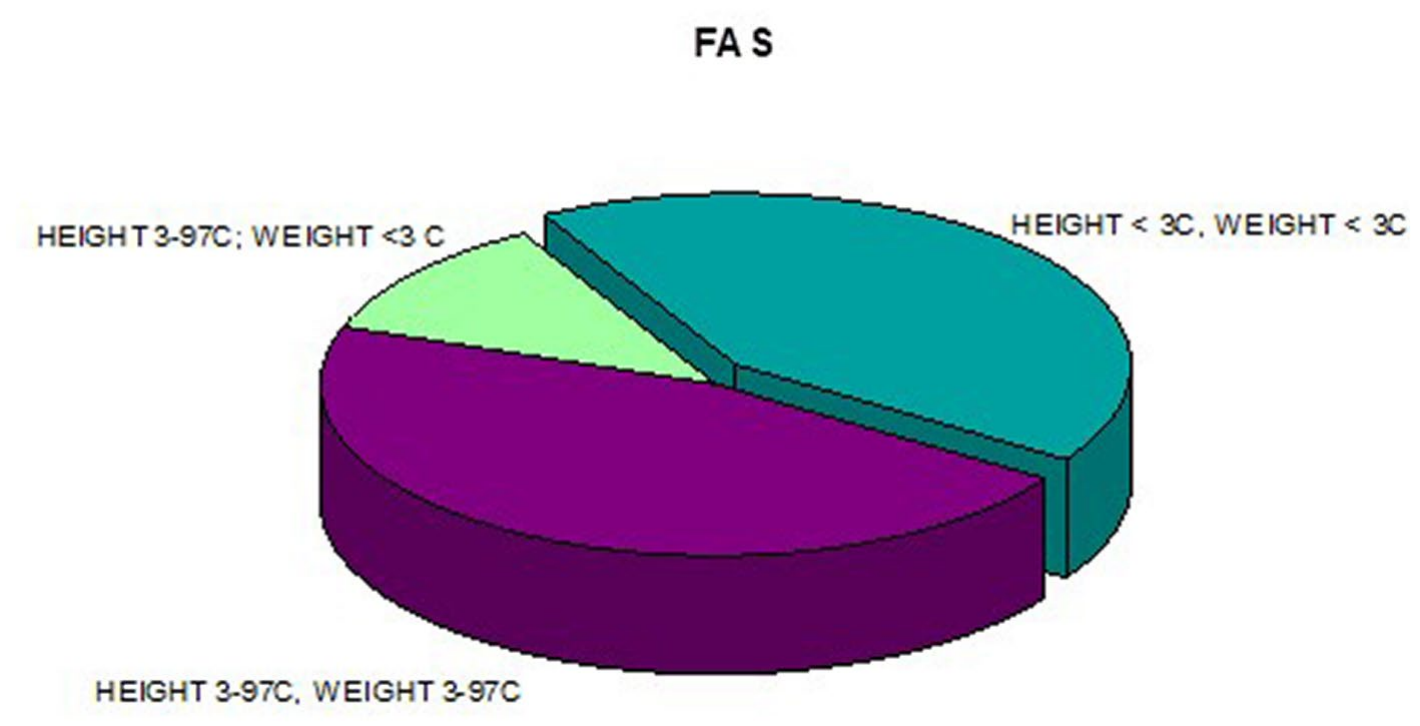
Co-occurrence of weight and height deficiency in patients with FAS.

**Figure 12:**
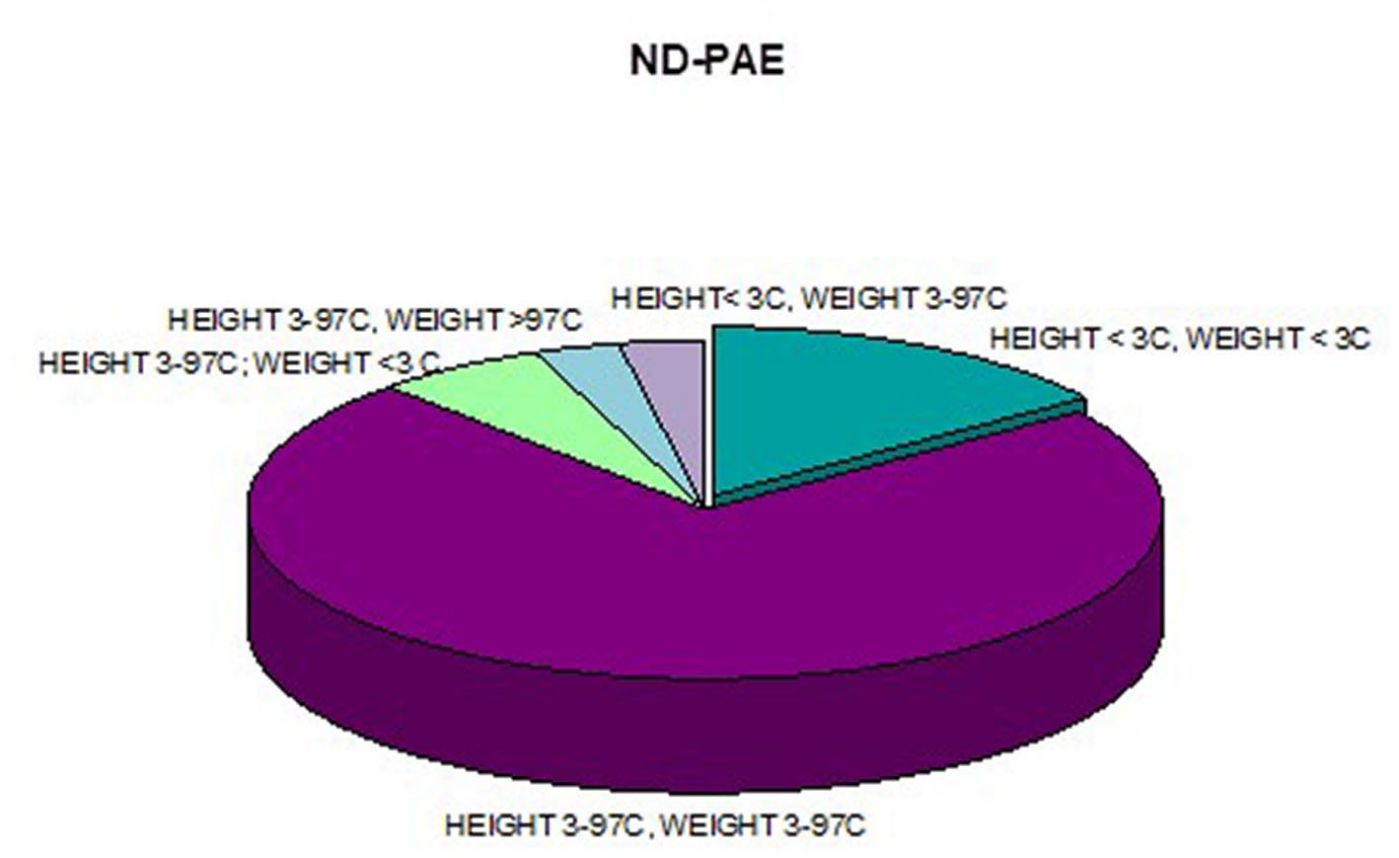
Co-occurrence of weight and height deficiency in patients with ND-PAE.

In addition, a combined analysis of body weight and height and the co-occurrence of low body height and underweight was presented in Figures 9–12. The prevalence of low body weight and short stature (both parameters <3rd centile) was found to be 27.11% in the whole group, in the individual groups: FAS – 42.30%, ND-PAE – 15.15% (Table 6). Normal body weight and height (both parameters in the 3 – 97th centile range) were found in 62.71% of the subjects and in the individual groups FAS – 46.15%, ND-PAE – 75.76%.

**Table 6:** Co-occurrence of low body height and short stature in subsequent groups FAS and ND-PAE.

	Height percentile	Weight percentile	Count/Whole group	% cooccurrence
FAS	**<3**	**<3**	11/26	**42.30%**
ND-PAE	**<3**	**<3**	5/33	**15.15%**
FASD	**<3**	**<3**	16/59	**27.11%**
FAS	3–97	3–97	12/26	46.15%
ND-PAE	3–97	3–97	25/33	75.76%
FAS	3–97	<3	3/26	11.54%
ND-PAE	3–97	<3	2/33	6.06%
ND-PAE	<3	3–97	1/33	3.03%

values below the 3rd percentile (height deficiency) are in bold.

**Figure 13:**
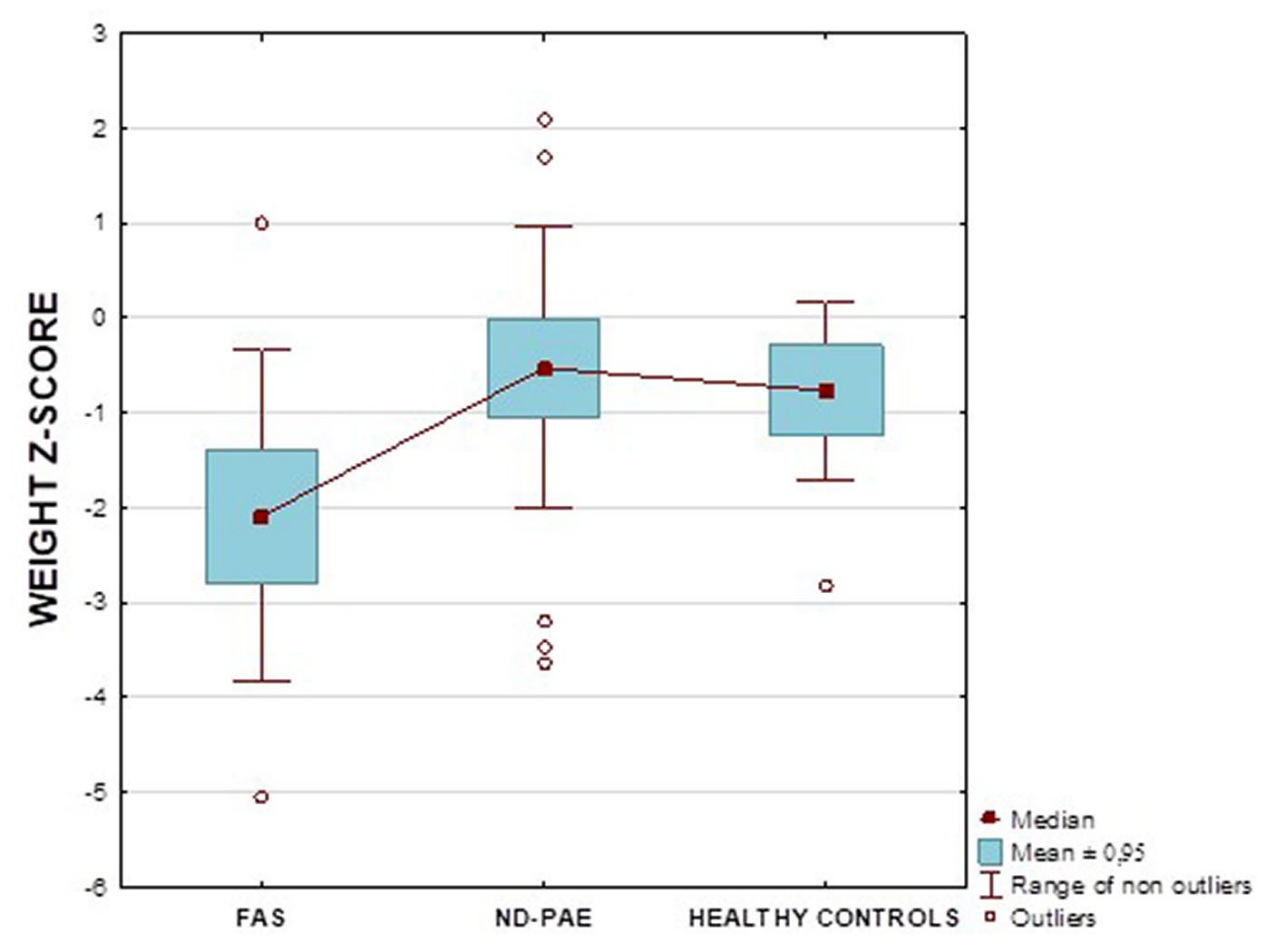
Weight *z*-score in patients with FAS and ND-PAE compared to healthy participants.

Figure 13 shows the weight *z*-score relationships between the FASD subgroups and healthy controls.

### 3.3. BMI results

In the FASD group, the mean value of the BMI percentile was 31.9 kg /m^2^ ± 27.48. The lower mean BMI values were related to the FAS group (21.71 kg/m^2^, median 12 kg/m^2^) compared to the ND-PAE group (39.62 kg/m^2^), differences were statistically significant (*p* = 0.03).

**Figure 14:**
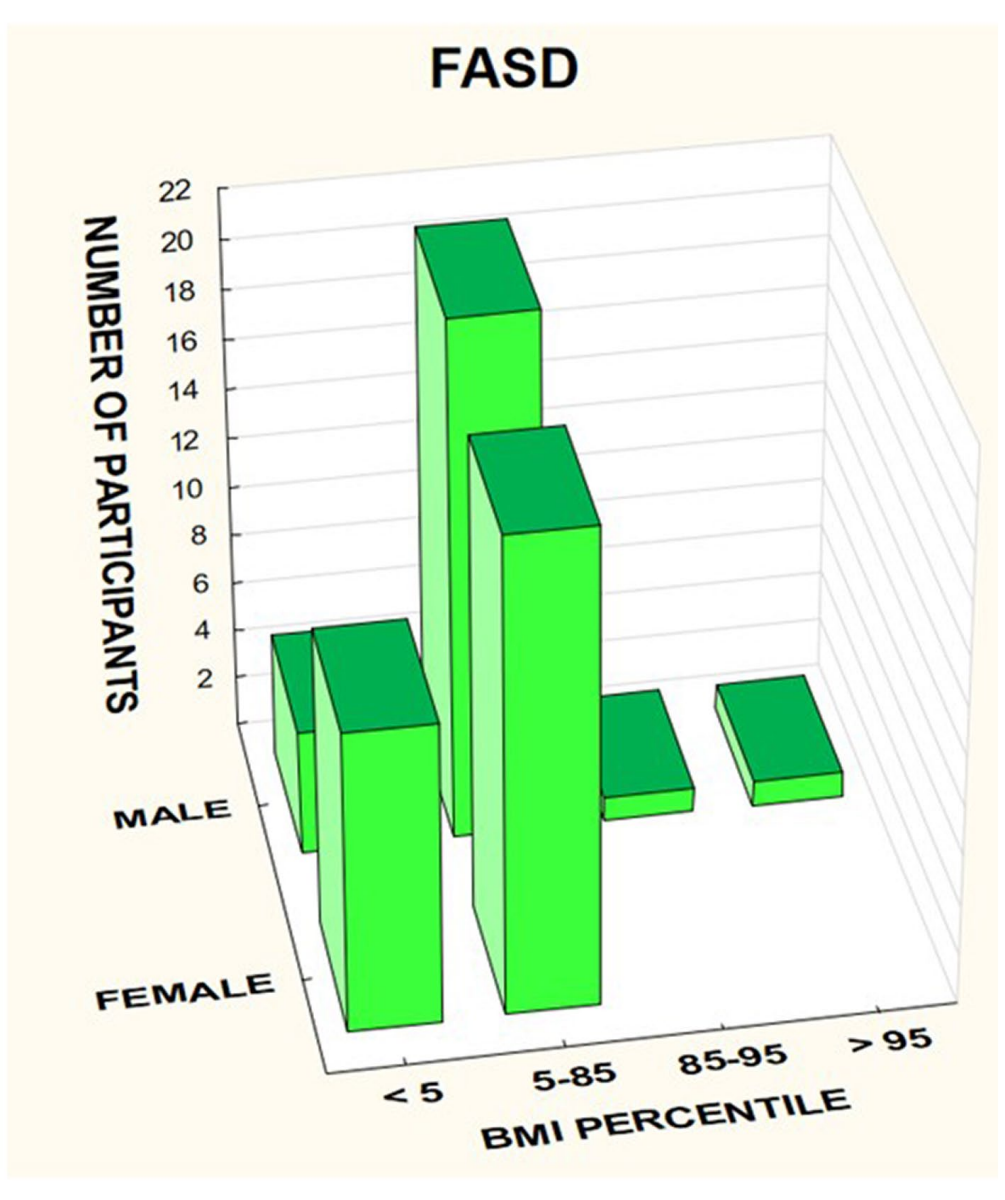
BMI percentile in relation to gender in patients with FASD.

**Figure 15:**
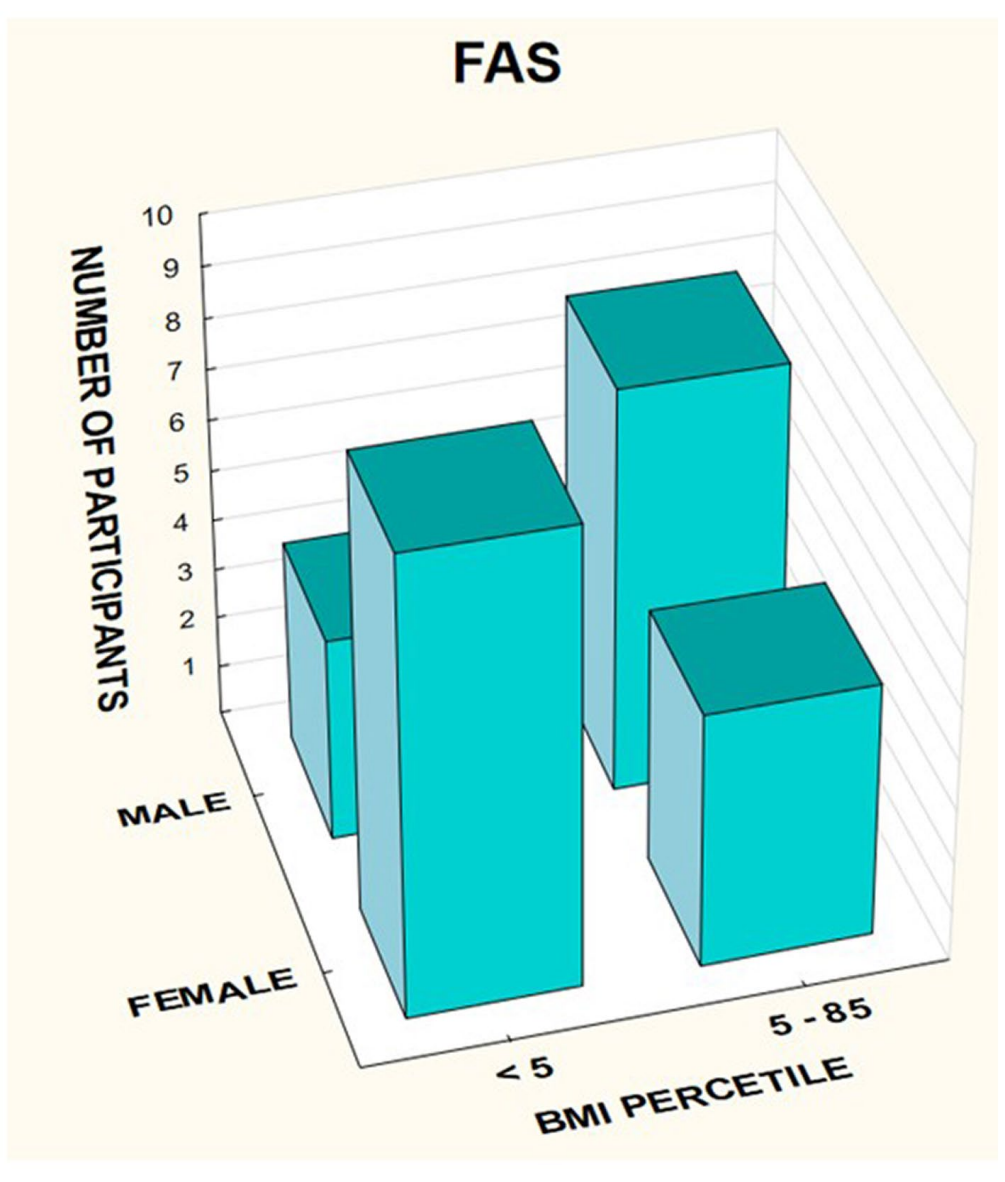
BMI percentile in relation to gender in patients with FAS.

**Figure 16:**
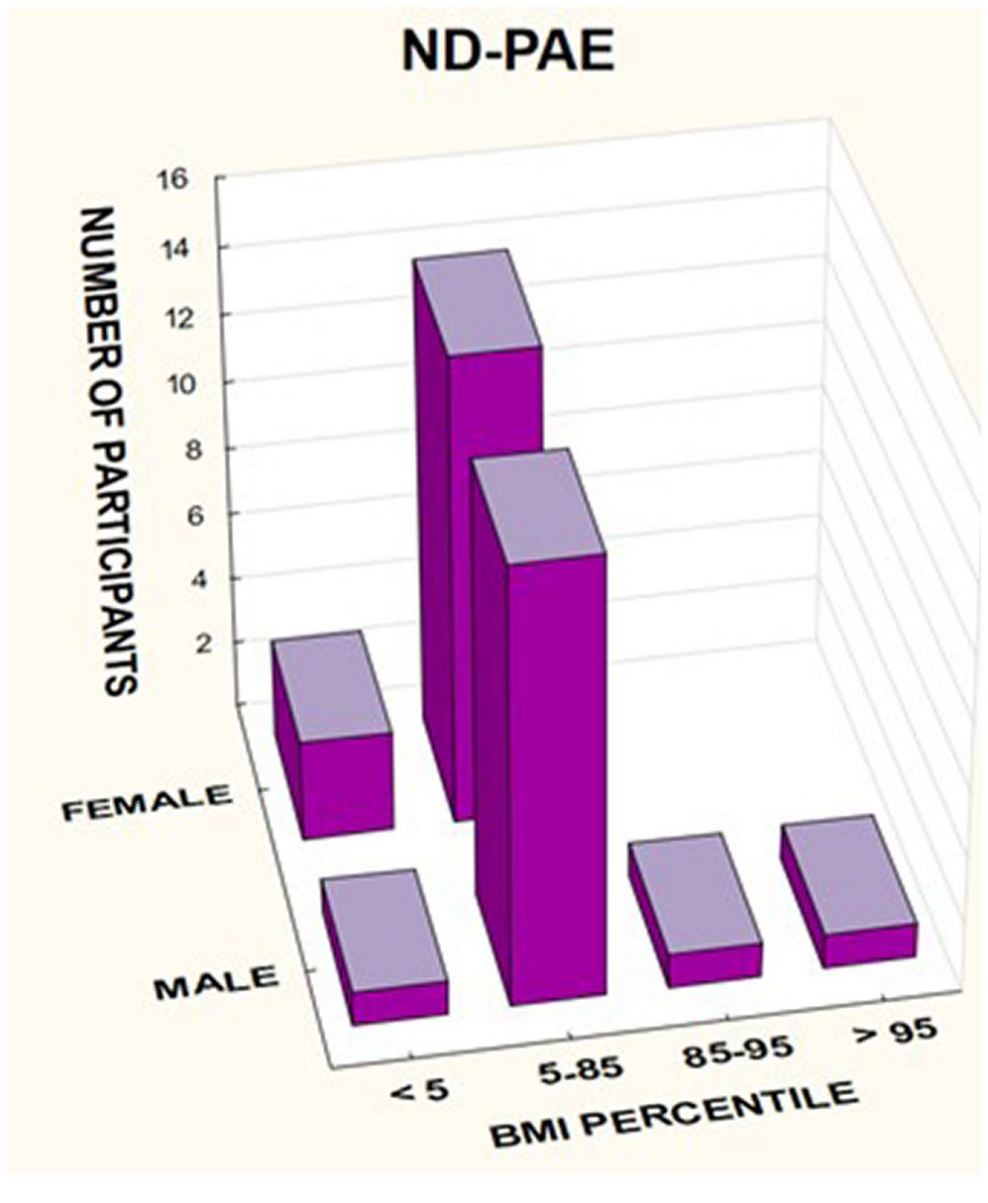
BMI percentile in relation to gender in patients with ND-PAE.

The BMI was then analyzed taking into account the division into gender and diagnosis (Figures 14–16).

In the group of children diagnosed with FAS, a low BMI below the 5th percentile affected 64.29% of girls, compared to 33.33% of boys. In this group, no indicator above 85% was found, regardless of gender (Table 7).

**Table 7:** BMI percentiles in subsequent groups FASD, FAS, and ND-PAE.

Study group	Gender	Percentile	Count	%
FAS *N*1 = 26	**Female**	**<5**	**9**	**64.29%**
	*N* = 10	May-85	5	35.71%
	**Male**	**<5**	**4**	**33.33%**
	*N* = 8	May-85	8	66.67%
ND-PAE *N*2 = 33	**Female**	**<5**	**3**	**17.65%**
	***N* = 17**	May-85	14	82.35%
	**Male**	**<5**	**1**	**6.25%**
	***N* = 16**	May-85	13	81.25%
		85–95	1	6.25%
		>95	1	6.25%
FASD *N* = 59		**<5**	**17**	**28.81%**
		May-85	40	67.80%
		85–95	1	1.69%
		>95	1	1.69%

values below the 5th percentile (underweight) are in bold.

**Figure 17:**
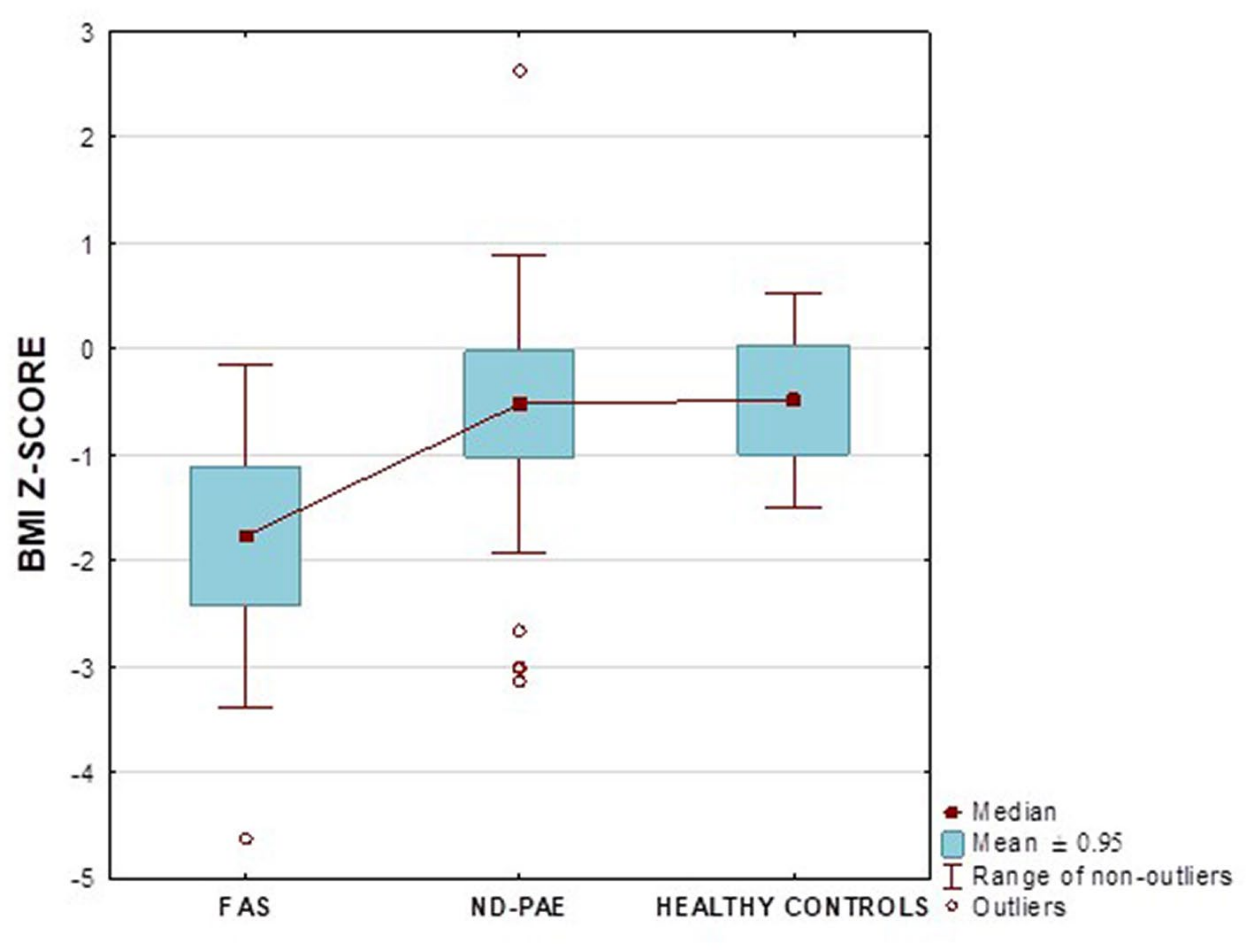
BMI *z*-score in patients with FAS and ND-PAE compared to healthy participants.

Figure 17 shows the BMI *z*-score relationships between the FASD subgroups and healthy controls.

In the group of children diagnosed with ND-PAE, the indicator below the 5th percentile was found in 17.65% of girls, compared to 6.25% of boys. Normal body weight (BMI 5–85%) was found in 82.35% of girls and 81.25% of boys. In this group, overweight was found in 6.25% of boys and obesity in 6.25% of boys.

A significant proportion of children with a low BMI below the fifth percentile were observed in the FAS group compared to the ND-PAE group; 50.0% vs. 12.12%.

**Figure 18:**
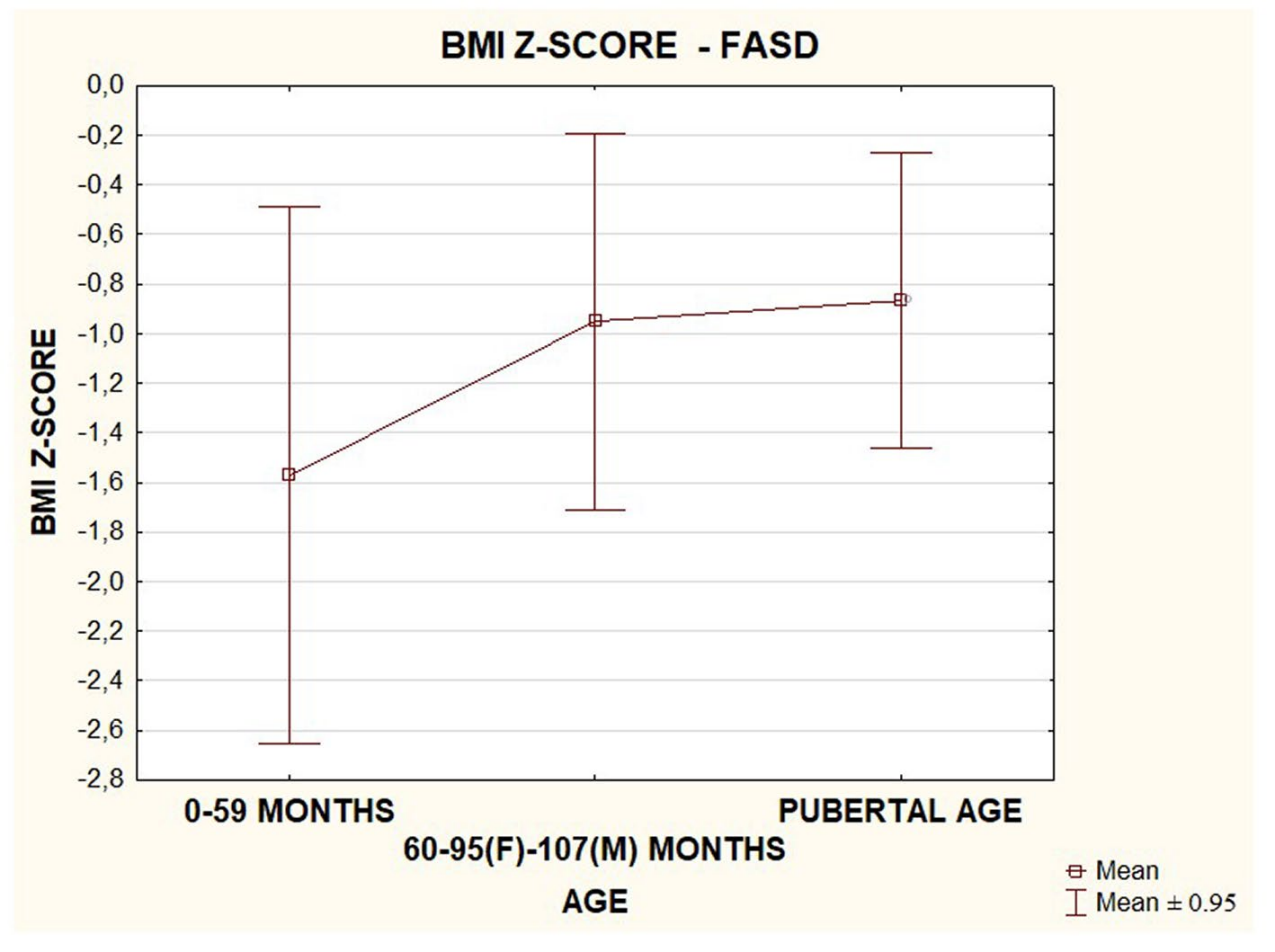
BMI *z*-score in relation to age in patients with FASD (F, female; M, male).

In the FASD group studied, BMI values were found to increase with the age of the patients. The data are presented in the Figure 18.

### 3.4. IGF-1 results

Fifty-two valid IGF-1 values were obtained in the study group. The mean value of IGF-1 was 165.8 ± 100.99 ng/mL. The median value of the calculated index: IGF-1/mean normal IGF-1 in the study group was 0.8120 ng/mL; IQR = 0.3943.

When comparing the IGF-1 index/mean normal IGF-1 values between patients in the FAS group and the ND-PAE group, there were no statistically significant differences between the groups (*p* = 0.61).

Based on the calculated IGF-1 index, there was a median and mean incidence of below average IGF-1 scores in the whole group, and in the FAS and ND-PAE subgroups (values at the lower limit of normal). Lower values were observed in the FAS group relative to the ND-PAE group. There was also a correlation between height centile and IGF-1 ratio/mean IGF-1 norm in the entire group. Lower IGF-1 values were found in children in the lower centile ranges.

Analyses on the effect of puberty on IGF-1 levels were also carried out in the children in each group. The participants in the different groups were divided according to pubertal stage (Tanner 1 versus Tanner 2–5). In the FAS group, statistically significant differences were found between prepubertal and pubertal patients (*p* = 0.002). No statistically significant differences were found in the other groups. The results are presented in Table 8.

**Table 8:** Comparison between prepubertal and pubertal participants according to IGF-1 level.

Pubertal status		Tanner stage 1	Tanner stage 2–5	*p* value
FAS		18/26	August-26	
*N*1 = 26	Mean ± SD	106.96 ± 42.94	252.88 ± 91.15	**0.002**
	Range	28.3–184	81–372	
ND-PAE		22/33	November-33	
*N*2 = 33	Mean ± SD	141.17 ± 63.29	241.22 ± 151.73	0.08
	Range	71.2–334	103–481	
FASD		40/56	19/26	
*N* = 59	Mean ± SD	126.51 ± 57.38	246.71 ± 123.21	0.16
	Range	28.3–334	81–481	
Healthy controls		16/23	July-23	
*N* = 23	Mean ± SD	110.14 ± 66.01	278.5 ± 120.35	0.06
	Range	53.4–217	123–397	

Statistically significant differences are bolded.

### 3.5. Correlations

The whole group showed a correlation between body weight, height and BMI *z*-score percentile and the IGF-1 index and weight and BMI with IGF-1 (significant positive correlations in Spearman rank order, Table 9). In the FAS group, no correlations between height, weight, and IGF-1 index were found, except positive correlation between IGF-1 ang BMI *z*-score (Spearman rank order correlations, Table 9). In the ND-PAE group, correlations were observed between height, weight ang BMI *z*-score and between weight and BMI *z*-score and IGF-1 (Spearman rank-order correlations, Table 9).

**Table 9:** Spearman correlation rank order between anthropometric parameters and IGF-1.

Group		Height *z*-score	Weight *z*-score	BMI *z*-score	IGF-1 [ng/mL]	IGF-1 index
**FASD *N* = 59**	Height *z*-score		*R* = 0.803*	*R* = 0.445*	*R* = 0.191	*R* = 0.440*
			***p* < 0.001**	***p* < 0.001**	*p* = 0.175	***p* = 0.001**
	Weight *z*-score	*R* = 0.803*		*R* = 0.848*	*R* = 0.393*	*R* = 0.446*
		***p* < 0.001**		***p* < 0.001**	***p* = 0.004**	***p* < 0.001**
	BMI *z*-score	*R* = 0.445*	*R* = 0.848*		*R* = 0.445*	*R* = 0.337*
		***p* < 0.001**	***p* < 0.001**		***p* < 0.001**	***p* = 0.015**
**FAS N1 = 26**	Height *z*-score		*R* = 0.811*	*R* = 0.284	*R* = −0.036	*R* = 0.122
			***p* < 0.001**	*p* = 0.159	*p* = 0.871	*p* = 0.581
	Weight *z*-score	*R* = 0.811*		*R* = 0,678*	*R* = 0,306	*R* = 0,255
		***p* < 0.001**		***p* < 0.001**	*p* = 0.156	*p* = 0.240
	BMI *z*-score	*R* = 0.284	*R* = 0.678*		*R* = 0.414*	*R* = 0,275
		*p* = 0.159	***p* < 0.001**		***p* = 0.049**	*p* = 0.205
**ND-PAE N2 = 33**	Height *z*-score		*R* = 0.758*	*R* = 0.361*	*R* = 0.351	*R* = 0.657*
			***p* < 0.01**	***p* = 0.039**	*p* = 0.062	***p* < 0.001**
	Weight *z*-score	*R* = 0.758*		*R* = 0.846*	*R* = 0.444*	*R* = 0.543*
		***p* < 0.001**		***p* < 0.001**	***p* = 0.016**	***p* = 0.002**
	BMI *z*-score	*R* = 0.361*	*R* = 0.846*		*R* = 0.445*	*R* = 0.336
		***p* = 0.039**	***p* < 0.001**		***p* = 0.016**	*p* = 0.075
**Healthy controls *N* = 23**	Height *z*-score		*R* = 0.689*	*R* = −0.009	*R* = −0,115	*R* = −0,333
			***p* = 0.002**	*p* = 0.973	*p* = 0.751	*p* = 0.381
	Weight *z*-score	*R* = 0.689*		*R* = 0.644*	*R* = 0.333	*R* = 0.283
		***p* = 0.002**		***p* = 0.005**	*p* = 0.347	*p* = 0.460
	BMI *z*-score	*R* = −0,009	*R* = 0,644*		*R* = 0.515	*R* = 0.417
		*p* = 0.973	***p* = 0.005**		*p* = 0.128	*p* = 0.265

^*^R with *p* < 0.05 are in bold.

## 4. Discussion

The problem of alcoholism in the modern world is a growing and unresolved phenomenon. Unfortunately, it is also reflected in the prevalence of spectrum disorders of FASD (22, 23) and affects all societies. From the point of view of pediatric endocrinology and diabetology, the main goals of care for these patients are to improve the rate of growth, increase the final height in adulthood, provide an appropriate diet to prevent possible excessive weight gain, especially during adolescence, observe sexual maturation, and provide continuous metabolic evaluation for carbohydrate and lipid metabolism (24, 25). The endocrine problems of patients with FASD are most often growth and weight deficiency (26) and pubertal disorders. One of the causes of short stature in this group of patients may be low birth weight relative to fetal age (27).

According to UNICEF, 14.6% of babies worldwide are born with low birth weight (28). Small for gestational age (SGA) is a newborn’s weight below – 2 SDS (18, 19). According to a study by Ouellette et al. up to 27% of children of mothers who drink during pregnancy are born with SGA (most among mothers who consume large amounts of alcohol) (29). Birth weight below the 10th percentile (in addition to low current body weight and growth deficiency) is one of the criteria for the diagnosis of full-blown fetal alcohol syndrome (FAS) in the recommendations of various expert groups, including Polish recommendations (8, 9). In Poland, there is a program to treat children with SGA with growth hormone, and among them, patients with FASD. The finding of height or weight deficiency during the diagnosis of these patients with FASD (doctor and psychologist), it is necessary to refer them to specialized clinics. A detailed differential diagnosis of these disorders should then be performed to exclude or confirm comorbidities and treat them (including hypothyroidism, hypopituitarism of somatotropin, premature or early puberty with a poor growth prognosis, celiac disease, autoimmune diseases, emotional deprivation, poor nutrition, vitamin deficiencies, congenital heart and kidney defects). In the group I studied, most of the patients were under the care of foster families or care institutions. However, it should be noted that many children with FASD remain undiagnosed or misdiagnosed and do not receive proper medical care. More research is needed in larger groups of patients and dissemination of knowledge on FASD is needed.

Given these problems, our study attempted to characterize the patients under the care of the Pediatric Endocrinology Clinic and the Outpatient Clinic. Children presented for diagnosis of FASD (evaluation by a pediatrician). Anthropometric and laboratory parameters were assessed, these children are still under our care and have tests performed appropriate to their condition and needs, and are also referred for specialized consultations. Few data in the literature on endocrine problems in this population, most of which are animal studies. Most of them refer to anthropometric measurements only in the context of making a diagnosis of FASD. It should be recalled that low height and weight (current and birth) are its components, which explains this approach.

The most readily available parameters that can be compared are percentiles, body mass index, and *z*-score by age. In cross-sectional studies in populations in the United States, Italy, and South Africa, children diagnosed with FAS and PFAS (similar to Polish ND-PAE) were found to have lower height, weight, and head circumference compared to controls (30). According to Łączmańska et al., the growth of children with FASD is delayed compared to the population of healthy children. Low body weight and shorter length at birth are also more common. However, a BMI below the third percentile is observed in 22% of children with FAS, with a frequency of 3% in healthy children (31).

In our study group, a body mass index below the fifth percentile (indicating weight deficiency) was present in 46.15% in the FAS group and 12.12% in the ND-PAE group. When examining the relationships between the FASD study group and the FAS and ND-PAE subgroups and the control group, statistically significant differences were obtained between the BMI percentiles, which correlates with the previous experience of the researchers. When performing a combined analysis of weight and height and the co-occurrence of their disorders, the prevalence of low body weight and short stature (both parameters <3rd percentile) in the entire group was found to be 27.11%. In individual groups, N1–17.19%: N2–7.81%. Similarly, a study by Klug et al. found that children with FAS had lower height and weight, while children with partial FAS had higher mean BMIs for comparison. However, no significant differences were found between the male and female sexes, with age-related differences: older children had higher BMIs. In the diagnostic process of FASD, the usefulness of body height measurement is significant in less than half of children with FAS and less than 30% with partial FAS (pFAS) (32). Due to the lack of a worldwide diagnostic consensus and the authors’ use of different classifications of FASD, it is not possible to compare the prevalence of reduced BMI values with our analyses.

A study by Astley et al. (14) found that height deficiency was as common as other major features of FASD. 19% of the study population had a height below the third percentile and 33% had a height below the 10th percentile. The height below the 10th percentile occurs in the FASD spectrum with increasing frequency along with the severity of other characteristics of FASD. According to Astley et al., the deficiency in length/height and weight varies with the child’s age. At birth, the percentile for weight is lower than for length. In contrast, at later ages, the deficiency is more about height. The weight percentiles increase gradually with age, while the height percentiles decrease gradually with age. Similar observations apply to male and female populations. According to this study, the severity of microcephaly correlates with height deficiency.

In our study group of children with FAS, body height below the 10th percentile was found in as many as 73.08% and below the 3rd percentile in 53.85%. In the group of children with ND-PAE, body height below the 10th percentile was found to be 36.36% and below the 3rd percentile at 21.21%. As in the literature, the prevalence of low height in the study population increases with the severity of the diagnostic features of FAS (dysmorphia present, neurodevelopmental disorders).

However, in a study by Astley et al., height deficiency was found as a characteristic of FASD in more than 50%, of which 19% of the patients were below the third percentile (low height according to Polish norms) and 33% below 10% (children who require observation of growth rate). Of the children with short stature (below the 3rd percentile), 19% had characteristic dysmorphia and 18% had neurological disorders (14). The prevalence of growth deficiency was also observed to be correlated with the severity of the diagnosis: 100% of children with FAS had a short stature, while those with pFAS had only 46%. The growth trajectory was altered. Growth centiles were shown to decrease with age, while weight centiles increased (excessive weight gain occurs during the “catch-up” period of growth and puberty). Prenatal alcohol exposure correlates with preterm birth (less than 37 Hbd) and postnatal low body length, and prenatal nicotine exposure correlates with low birth weight. Growth deficiency is strongly correlated with the severity of neurological problems and the presence of dysmorphia (33).

In our study, low stature was found in a high percentage compared to data from the literature. This difference may be due to the number of children studied and the way in which the analyzed group was selected, and the children may also have been referred more often to counseling centers due to their lower height, which requires specialized management.

Most analyses of children with FASD are concerned with body height, whereas little attention has been paid to the prevalence of overweight and obesity in this population. A study by Brix et al. (34) described 445 children with FASD and 171 without this diagnosis, aged 2–19 years. 34% of the FASD group were obese or overweight, which did not differ from the group without FASD and the prevalence in the population. Low body weight was common in children with FAS (17%). In the entire group, there was a partial FAS (may partially correspond to the Polish diagnosis of ND-PAE), in which an increased prevalence of overweight/obesity was observed, 40%. In adolescents, excess body weight was observed more often in girls (50%) versus all adolescents (42%).

The prevalence of overweight and obesity in adolescents was three times higher than the population rate. According to the study authors, the results suggest that exposure to alcohol is a risk factor for endocrine and metabolic disorders. This condition may require targeted intervention, including diet intervention in prepubertal girls (34). Similar results were obtained in a study by Hayes et al. (35), which found that significant prenatal exposure to ethanol results in an increased risk of overweight and obesity in adolescent girls (35). In our study, no differences in BMI were observed when comparing children with FASD with respect to pubertal stage. The lack of prevalence of overweight and obesity may be due to proper care of children by caregivers, their education about the use of diet, and adequate physical activity. A limitation for future comparisons is the small size of the study group.

Height, weight, and BMI are correlated with insulin-like growth factor levels, among other factors. Although the growth hormone exerts some effects by acting directly on target tissues, other effects are exerted by insulin-like growth factors (IGFs). In adults, there are 2 forms: IGF-1 and IGF-2, originally produced in the liver. These growth factors, whose production is induced by HGH, interact with tissues, exerting an indirect growth hormone effect (36). Furthermore, insulin-like growth factors during pregnancy play an important role in the regulation of fetal growth and development (37), thus affecting the size of birth weight and therefore often the subsequent postnatal growth trajectory. In the studies conducted, no alcohol-induced changes in growth hormone action and secretion have been found in humans. Several animal studies have shown that ethanol consumption can affect IGF levels in pregnant individuals, and reductions in IGF-binding proteins have been reported. Other studies in animal models have shown that newborns had reduced levels of GH after exposure and that young animals had reduced amounts of IGF-1 in their blood (38–40).

Under normal conditions, growth hormone is released from the pituitary gland periodically throughout the day. GH secretion is modulated by various factors, such as changes in blood glucose and insulin levels, starvation, or physical activity (41). In human studies, it has been shown that in children with FASD, the response of GH to stimulants and during the sleep period is within normal limits, but the level of spontaneously secreted growth hormone was reduced (42, 43), so a reduction in IGF-1 levels is also expected. IGF-1 secretion increases with age and peaks during puberty and growth acceleration (44), which is reflected in laboratory standards. According to a study by Boguszewski et al., about 60% of children with short stature born with SGA have reduced spontaneous growth hormone (HGH) secretion in a post-sleep test and/or low HGH secretion in stimulation tests (44).

According to a study by Cutfield et al., some children with low birth weight have normal or high levels of IGF-1, suggesting the presence of resistance to insulin-like growth factor (45). In children with FASD, low growth often coexists with low body weight, which in healthy children often results in lower levels of IGF-1. It is not known what causes the potential reduction in IGF-1 levels in this group of patients, while Iwayama’s studies have shown the poor utility of IGF-1 as a single predictor of growth hormone deficiency (46). According to Caregaro et al., insulin-like growth factor-1 represents a biochemical marker of malnutrition in patients with eating disorders (47).

In a study by Andreu-Fernández et al., the analysis of serum IGF-1 and IGF-2 concentrations showed that children with FASD (and other prenatal alcohol exposure), showed significantly lower concentrations of both IGF-1 and IFG-2 than the control group and reference values. According to these authors, these results (positive Spearman correlations of anthropometry with IGF-1) support the use of growth factors IGF-1 and IGF-2 as surrogate biomarkers of damage caused by prenatal exposure to ethanol and can be used in the diagnosis of fetal alcohol disorders (48).

In contrast, our study analyzed the correlations between standardized *z*-scores for height, weight, and BMI with serum IGF-1 levels and the calculated IGF-1 index. During the study analyses, to make the results comparable regardless of age, the IGF-1 index was calculated, which is the quotient of the IGF-1 level and the average norm for age. The results confirm the correlation between weight, BMI and IGF-1 levels in the FASD, FAS and ND-PAE groups. Surprisingly, such correlations were not obtained in the control group (limited by the small number of results). It would be advisable to determine the levels of these proteins in a larger group of patients for possible use as a marker of growth disorders in patients with FASD.

Limitations of the study I conducted are mainly the sample size of patients with FASD (59 children), difficulties in obtaining consent for the study, lack of consideration of the influence of the family caring for the child, the therapies used, and developmental difficulties on the child’s nutritional status. Children with FASD exhibit difficulties with sensory hypersensitivity, have increased appetite for sweet foods, sometimes significant food selectivity, and impaired appetite regulation, which can affect their nutritional status in various ways. Further studies are needed in larger groups of patients taking into account hormonal factors.

## Data availability statement

The raw data supporting the conclusions of this article will be made available by the authors, without undue reservation.

## Ethics statement

The studies involving human participants were reviewed and approved by the Ethics Committee of the University of Rzeszow (date of approval: February 16, 2019). Written informed consent to participate in this study was provided by the participants’ legal guardian/next of kin.

## Author contributions

AD and AM: conceptualization, methodology, and writing—review and editing. AD: formal analysis, investigation, data curation, and writing—original draft preparation. All authors have read and agreed to the published version of the manuscript.

## Conflict of interest

The authors declare that the research was conducted in the absence of any commercial or financial relationships that could be construed as a potential conflict of interest.

## Publisher’s note

All claims expressed in this article are solely those of the authors and do not necessarily represent those of their affiliated organizations, or those of the publisher, the editors and the reviewers. Any product that may be evaluated in this article, or claim that may be made by its manufacturer, is not guaranteed or endorsed by the publisher.
